# Supplementary material for: Fate of antibiotic resistant E. coli and antibiotic resistance genes during full scale conventional and advanced anaerobic digestion of sewage sludge
Source: PLoS One. 2020 Dec 1;15(12):e0237283. doi: 10.1371/journal.pone.0237283 (PMC7707479; doi:10.1371/journal.pone.0237283)
Supplement: S1 Table — Table shows primer name and sequence, length (in base pairs) of amplicon, annealing temperature, sources, and (where relevant) which antibiotic the target gene confers resistance to. (DOCX) [file pone.0237283.s001.docx]

**S1 Table**

|  | Sequence | Amplicon Size | Annealing Temperature (˚C) | Reference | Confers Resistance to |
| --- | --- | --- | --- | --- | --- |
| 16S 967 -FW | CAACGCGAAGAACCTTACC | 98 | 60 | (Huse et al., 2008) | N/A |
| 16S 1046 -RV | AGGTGNTGCATGGCTGTCG |  |  |  |  |
| *tetM -*FW | ACAGAAAGCTTATTATATAAC | 171 | 60 | (Aminov et al., 2001) | Tetracycline |
| *tetM -*RV | TGGCGTGTCTATGATGTTCAC |  |  |  |  |
| bla-CTX-M-1 Family -FW | ACCAACGATATCGCGGTGAT | 101 | 60 | (Colomer-Lluch et al., 2011) | β-lactam |
| bla-CTX-M-1 Family -RV | ACATCGCGACGGCTTTCT |  |  |  |  |
| bla-CTX-M-9 Family- FW | ACCAATGATATTGCGGTGAT | 85 | 60 | (Colomer-Lluch et al., 2011) | β-lactam |
| bla-CTX-M-9 Family- RV | CTGCGTTCTGTTGCGGCT |  |  |  |  |
| *bla-IMP* -FW | GGAATAGAGTGGCTTAAYTCTC | 188 | 60 | (Ellington et al., 2006) | Carbapenem |
| *bla-IMP* -RV | CCAAACYACTASGTTATCT |  |  |  |  |
| *qnrS –*FW | GGCATTGTTGGAAACTTGCA | 118 | 60 | (Colomer-Lluch et al., 2014) | Flouro-quinolone |
| *qnrS­* -RV | CGACGTGCTAACTTGCGTGA |  |  |  |  |
| *aac(3)-1* –FW | ACCTACTCCCAACATCAGCC | 158 | 60 | (Zhang et al., 2017) | Gentamycin |
| *aac(3)-1* –RV | ATATAGATCTCACTACGCGC |  |  |  |  |
| *dfrA1* –FW | CTGTTGGTTGGACGCAAGAC | 180 | 60 | This study | Trimethoprim |
| *dfrA1 –*RV | CCCACCACCTGAAACAATGAC |  |  |  |  |
| *dfrA5* –FW | ATCGAAGAGGCCATGTACGG | 96 | 60 | This study | Trimethoprim |
| *dfrA5* -RV | AGAGGCCATGGGCAATGTTT |  |  |  |  |
| *dfrA7* -FW | TCTGGTGGCGGTCAAATCTAC | 82 | 60 | This study | Trimethoprim |
| *dfrA7 -*RV | CTTCAACCTCAACGTGAACAGT |  |  |  |  |
| *dfrA12* –FW | CACGCTATCGCTTTGGCATC | 146 | 60 | This study | Trimethoprim |
| *dfrA12* -RV | ATTGGGAAGAAGGCGTCACC |  |  |  |  |
| *dfrA17 –*FW | TGGCGTAATCGGTAGTGGTC | 101 | 60 | This study | Trimethoprim |
| *dfrA17* -RV | TTCTTCCGACAAGGAGCCAT |  |  |  |  |
| *sul1* –FW | CGCACCGGAAACATCGCTGCAC | 163 | 60 | (Pei et al., 2006) | Sulphonamide |
| *sul1* -RV | TGAAGTTCCGCCGCAAGGCTCG |  |  |  |  |
| *ermF* –FW | TCGTTTTACGGGTCAGCACTT | 182 | 60 | (Schmidt et al., 2015) | Erythromycin |
| *ermF* -RV | CAACCAAAGCTGTGTCGTTT |  |  |  |  |
| *int1*- FW | CAGTGGACATAAGCCTGTTC | 160 | 60 | (Koeleman et al., 2001) | N/A |
| *int1-* RV | CCCGAGGCATAGACTGTA |  |  |  |  |

**S1 Table.** **Primers and annealing temperatures used in this study.** Table shows primer name and sequence, length (in base pairs) of amplicon, annealing temperature, sources, and (where relevant) which antibiotic the target gene confers resistance to.
