# Supplementary material for: Fate of antibiotic resistant E. coli and antibiotic resistance genes during full scale conventional and advanced anaerobic digestion of sewage sludge
Source: PLoS One. 2020 Dec 1;15(12):e0237283. doi: 10.1371/journal.pone.0237283 (PMC7707479; doi:10.1371/journal.pone.0237283)
Supplement: S2 Table — (DOCX) [file pone.0237283.s002.docx]

**S2 Table**

| Reagent | Volume added per reaction (µl) |
| --- | --- |
| SYBR Green | 7.5 |
| Forward and Reverse Primer Mix (10ng/µl) | 3 |
| DNA template (approx. 40ng/µl) | 3 |
| Nuclease Free Water | 1.5 |

**S2 Table.** **Reaction matrix used for qPCR of resistance genes**, *int1*, and the 16S rRNA gene.
