# Supplementary material for: Fate of antibiotic resistant E. coli and antibiotic resistance genes during full scale conventional and advanced anaerobic digestion of sewage sludge
Source: PLoS One. 2020 Dec 1;15(12):e0237283. doi: 10.1371/journal.pone.0237283 (PMC7707479; doi:10.1371/journal.pone.0237283)
Supplement: S1 Fig — The percentage of E. coli clinical and WWTP isolates resistant to each antibiotic tested in this study is shown. Clinical data from Public Health Wales (2017). (DOCX) [file pone.0237283.s003.docx]

**S1 Fig**


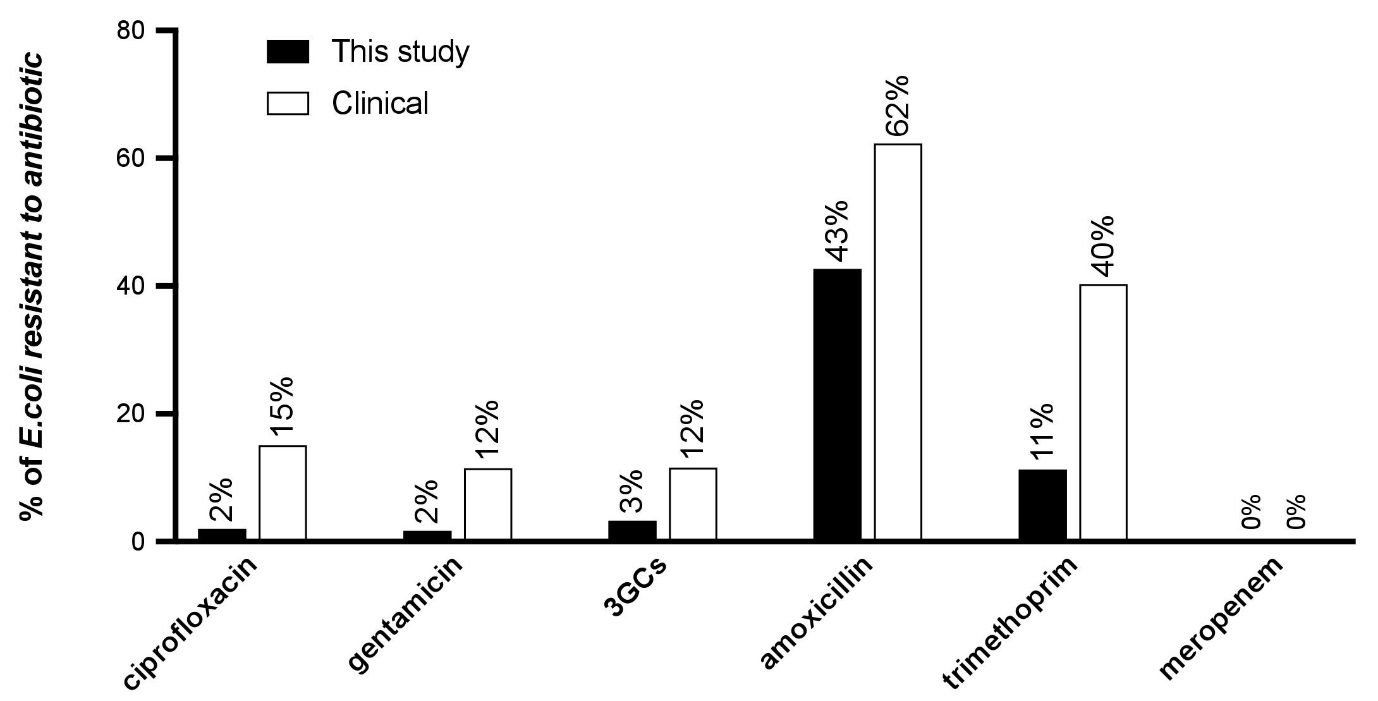


**S1 Fig.** **Comparison of resistance rates in clinical isolates with resistance rates in isolates from WWTP sludge**. The percentage of *E. coli* clinical and WWTP isolates resistant to each antibiotic tested in this study is shown. Clinical data from Public Health Wales (2017).
