# Supplementary material for: Fate of antibiotic resistant E. coli and antibiotic resistance genes during full scale conventional and advanced anaerobic digestion of sewage sludge
Source: PLoS One. 2020 Dec 1;15(12):e0237283. doi: 10.1371/journal.pone.0237283 (PMC7707479; doi:10.1371/journal.pone.0237283)
Supplement: S2 Fig — A. Before AD, WWTP 2. B. After AD, WWTP 2. No significant phylotype shift during AD was observed. (DOCX) [file pone.0237283.s004.docx]

**S2 Fig**


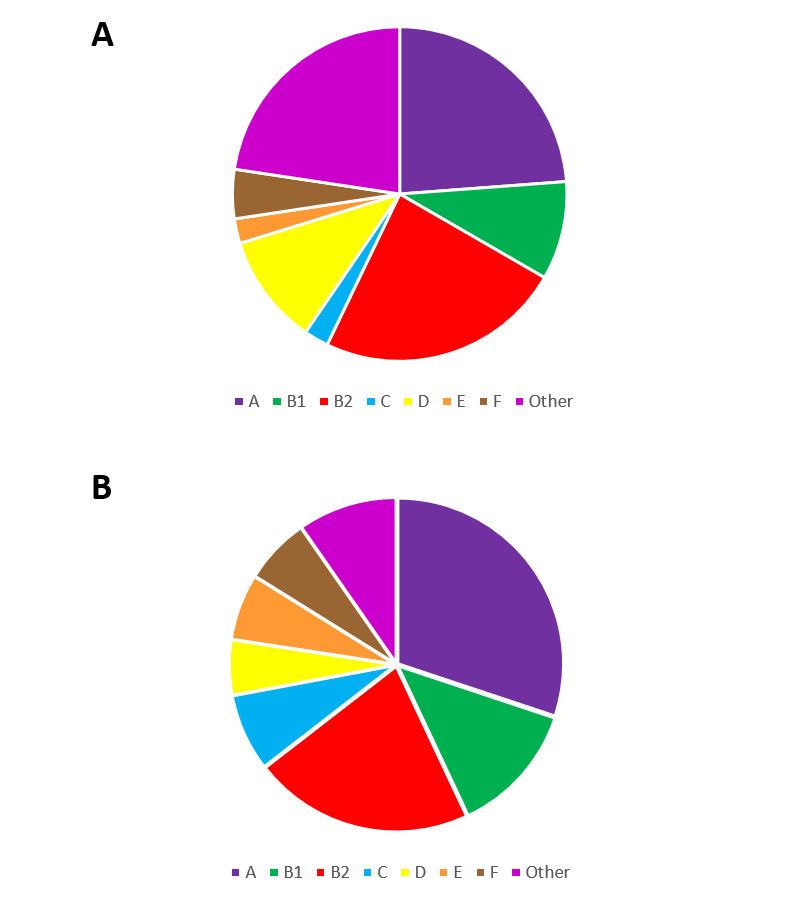


**S2 Fig. *E.coli* phylotypes**. **A**. Before AD, WWTP 2. **B.** After AD, WWTP 2. No significant phylotype shift during AD was observed.
