# Supplementary material for: Fate of antibiotic resistant E. coli and antibiotic resistance genes during full scale conventional and advanced anaerobic digestion of sewage sludge
Source: PLoS One. 2020 Dec 1;15(12):e0237283. doi: 10.1371/journal.pone.0237283 (PMC7707479; doi:10.1371/journal.pone.0237283)
Supplement: S3 Fig — There is 100% similarity between the sewage sludge sequence and the plasmid version of ermF. (DOCX) [file pone.0237283.s005.docx]

**S3 Fig**


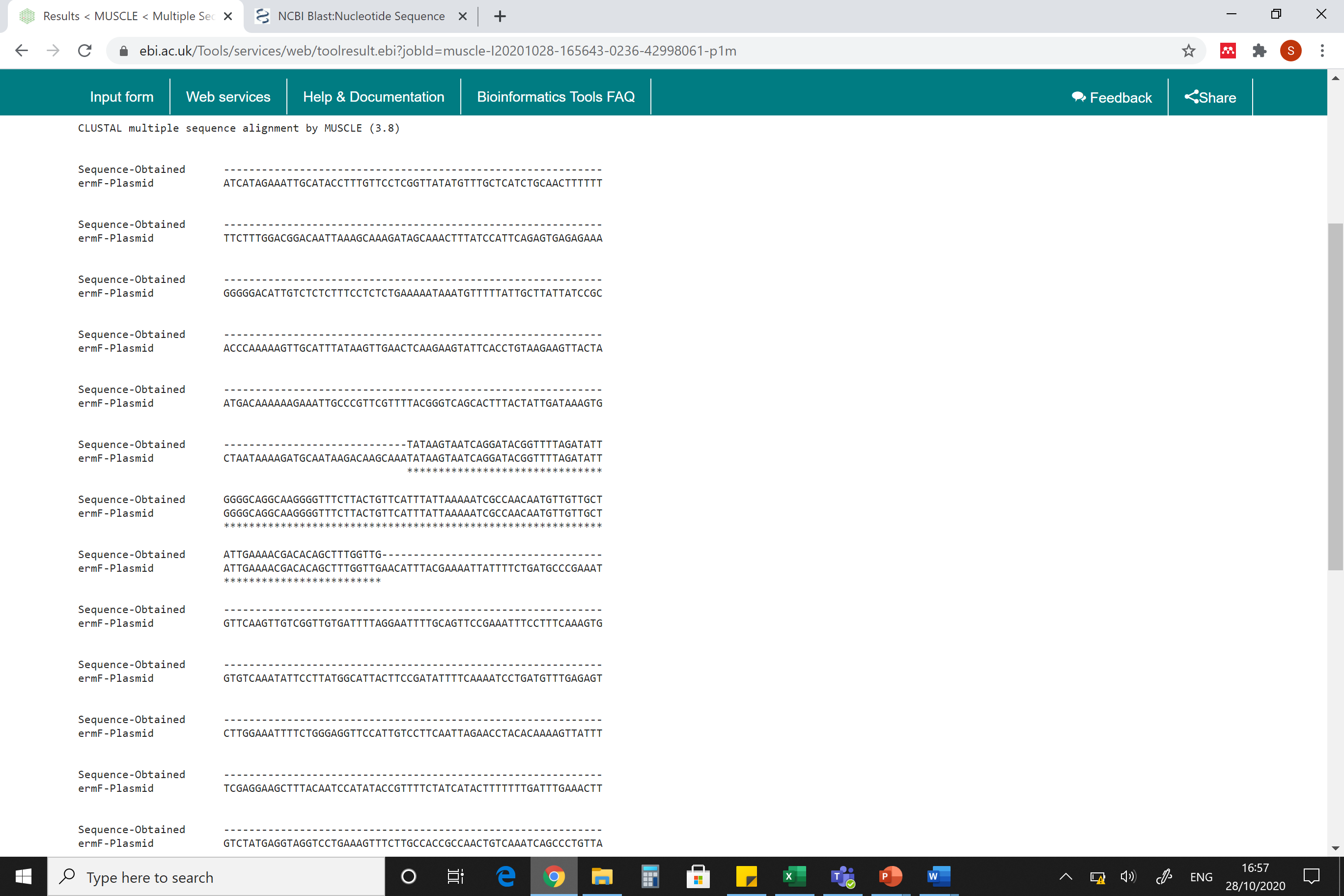


**S3 Fig. An alignment between the ermF sequence obtained from sewage sludge with a plasmid form of ermF from a clinical isolate of Bacteroides fragilis BLAST Sequence ID M14730.1.**  There is 100% similarity between the sewage sludge sequence and the plasmid version of ermF.
