# Supplementary material for: Fate of antibiotic resistant E. coli and antibiotic resistance genes during full scale conventional and advanced anaerobic digestion of sewage sludge
Source: PLoS One. 2020 Dec 1;15(12):e0237283. doi: 10.1371/journal.pone.0237283 (PMC7707479; doi:10.1371/journal.pone.0237283)
Supplement: S4 Fig — Graph shows before TH, after TH and after TH-MAD at WWTP1, as well as before and after MAD at WWTP2. (DOCX) [file pone.0237283.s006.docx]

**S4 Fig**

**S4 Fig. E. coli, other coliforms and non-coliform Gram negatives CFUs**. Graph shows before TH, after TH and after TH-MAD at WWTP1, as well as before and after MAD at WWTP2.
